# Prevalence of Falls and Its Associated Factors among Elderly Diabetes in a Tertiary Center, Malaysia

**DOI:** 10.1155/2012/539073

**Published:** 2012-05-30

**Authors:** A. K. Azidah, H. Hasniza, E. Zunaina

**Affiliations:** ^1^Department of Family Medicine, School of Medical Sciences, Universiti Sains Malaysia, 16150 Kubang Kerian, Malaysia; ^2^Department of Opthalmology, School of Medical Sciences, Universiti Sains Malaysia, 16150 Kubang Kerian, Malaysia

## Abstract

The purpose of this study is to determine the prevalence of falls and its associated factors among elderly diabetes type 2 patients attending a tertiary center in Malaysia. We conducted a cross-sectional study among 288 elderly diabetes type 2. The data collected includes data on sociodemographic, diabetes history, comorbid diseases, drug use, and activity of daily living (Barthel's index). The patient also was examined physically, and balance and gait assessment was carried out. Prevalence of falls among elderly diabetes was 18.8%. Female gender (OR: 2.54, *P* < 0.05), age group more than 75 (OR: 2.97, *P* < 0.05), retinopathy (OR: 2.19, *P* < 0.05), and orthostatic hypotension (OR: 2.87, *P* < 0.05) were associated with higher risk for falls. High balance and gait score was associated with reduced risk of fall in elderly diabetes (OR: 0.89, *P* < 0.05). In conclusion, the factors that are associated with higher risk for falls among elderly diabetes were female sex, age group more than 75, presence of retinopathy, and orthostatic hypotension. Those who had higher balance and gait score were found to be less likely to fall compared with those with lower score.

## 1. Introduction

The number of elderly people within a population is increasing worldwide especially in developing countries. Falls are a major problem in the elderly because they cause significant morbidity and mortality. This is due to complications arising from falls causing a significant decrease in functional status, serious injury, and increased utilization of medical services [1, 2]. Diabetes mellitus (DM) is highly prevalent in older people [3]. The current burden of diabetes is greatest in the population ≥65 years of age, where more than half of direct medical expenditures were on diabetes [4]. As diabetes increasingly becomes a disease of elderly people, some of its related complications must be addressed. These include cognitive disorders and physical disability, falls and fractures, and other geriatric syndromes in the elderly [5].

There is evidence that shows that diabetes mellitus is a predictor of falls risk [6–8]. A number of studies have reported that older people with diabetes have a higher risk of falls compared with those without diabetes [3, 8]. Diabetes-related complications such as peripheral neuropathy [9, 10], reduced vision [6], diabetic foot ulcers, and impaired renal function [11] are the potential mechanism for falls. Reduced balance, strength and gait, are also likely conditions associated with diabetes-related complications and risk of falls [6, 8, 12].

The consequences of falls are said to be more severe in the elderly with diabetes [12]. The reasons for this may include slower wound healing [13] delaying recovery from falls and a higher likelihood of fractures, a result of bone mineral [14] and strength loss with age [6].

Based on these reasons, it is expected that the prevalence of falls in the elderly with diabetes would be higher than the general elderly population. In comparison, the prevalence of falls in the general elderly community is about 30% [2]. However, the prevalence of falls has been reported to be as high as 78% in long-term care facility residents with diabetes [15] and 43.7% in those elderly with diabetes living in rural areas [12].

As far as we know, no reported study regarding falls in elderly diabetics in Asian countries has been yet found. Thus, it is important to assess the burden of this problem and to identify its associated factors for planning any intervention by the policymaker. The purpose of this study is to establish the prevalence of falls as well as to determine the falls risk associated with falls in elderly diabetics in Malaysia.

## 2. Subject and Methods

We conducted a cross-sectional study commencing from April, 2007 until March 30, 2008, among 288 elderly patients suffering from type 2 diabetes. This study was conducted in a tertiary center in the East Coast of Peninsular Malaysia (Hospital Universiti Sains Malaysia). Falls were defined as unintentionally coming to the ground or some lower surface and not as a consequence of sustaining a violent blow, loss of consciousness and sudden paralysis as in a stroke incident or epileptic seizure. In this study, falls were defined as having at least one history of falling in the past one year from the interview date, and frequent falls were defined as experiencing two or more falls in the past one year from the interview date. The elderly is defined as those over 60 years of age, adopting the criteria set at the World Assembly on Aging in Vienna in 1982 (Ministry of National Unity and Social Development, 1996). Nonambulatory patients and those unable to stand unassisted for a minimum of one minute were excluded from the study. Hypoglycemia episodes are defined as the presence of hypoglycemia in a person occurring within the past one year, based upon symptoms reported by patients, and orthostatic hypotension (OH) is defined as a reduction of systolic blood pressure (BP) of at least 20 mmHg, or diastolic BP of at least 10 mmHg with a transition from supine to standing position. Polypharmacy was defined as using four or more types of medications. The presence of retinopathy is defined upon the discovery of retinopathy either by fundus camera or ophthalmologist assessment. The presence of nephropathy was based upon positive urine microalbuminurmia on two occasions, three to six months apart, and serum creatinine levels reaching more than 133 mmol/L. The diagnosis of hypertension was defined by a self-report from a physician's diagnosis or as reported in the case note.

### 2.1. Data Collection

The subjects were identified during their regular diabetes clinical followup and were selected using a systematic random sampling method. The subjects were approached based on the ratio of 1 : 2 centered upon registration lists at the clinic. Written informed consent was obtained from the subjects or caretakers for the participations of the study.

Patients were given questionnaire on sociodemographic details to fill in which consists of questions to assess age, ethnicity, gender, marital status, educational status, occupational status, smoking status, and living arrangement. Then, physical examinations were performed on the subjects by the doctor. The physical examination includes measurement of height, weight, blood pressure during standing and sitting, monofilament testing, and Tinetti Balance and Gait Assessment [16]. Assessment of impaired foot sensation was documented objectively using a 5.07 Semmes-Weinstein monofilament, and the functional assessment was assessed using Barthel's index [17], and the usage of assistive devices were recorded. Review of patients's hospital records for medications usage, laboratory investigations results, and medical illnesses were done. Later, patients were referred to the ophthalmology clinic for proper visual assessment.

### 2.2. Statistical Analysis

Sample size calculation was done for all the objectives of the study, and the biggest sample size calculated was chosen for the study. The sample size chosen for this study is based on the sample size to determine the prevalence of fall among elderly diabetes which was calculated using the single proportion formula. By using the prevalence of fall 43% [12], taking the precision of 0.06 and considering nonresponse rate of rate 10%, the calculated sample size was 288.

All data was entered and analyzed using Statistical Program for Social Sciences (SPSS) version 12.0 (SPSS Inc., 2003). Simple logistic regression was used as a screening in selection of variables for further analysis. All variables with *P* value less than 0.25 and clinical variables were included in the multiple logistic regression analysis. The method that was used for variable selection was backward and forward stepwise procedure. All possible 2-way interactions were checked, and those significant variables were included in the model. The independent variables were fitted into multiple logistic regression, and variance inflation factors were obtained to check for multicollinearity. Fitness of model was tested by Hosmer-Lemeshow goodness of fit test, the classification table and receiver operator characteristic curve.

### 2.3. Approval by the Research and Ethics Committee

The protocol was approved by the Research and Ethical Committee, School of Medical Sciences, Universiti Sains Malaysia on March 22, 2007.

## 3. Results

### 3.1. Characteristics

A total 316 subjects that fulfilled the inclusion and exclusion criteria were approached during study period. However, only 288 subjects consented. Thus, the response rate of the study was 91.1%.  Table 1 shows the demographic and clinical characteristics of the study participants. The mean age of the study participant was 66.9 (5.81). Total balance and gait scores among faller group were lower compared to nonfaller. Hypertension, retinopathy, peripheral neuropathy, orthostatic hypotension, and polypharmacy and hypoglycemia episodes were more common in the fallers compared to nonfallers.

### 3.2. Prevalence of Falls and Its Characteristics

The prevalence of fall among elderly diabetes was 18.8%. Among the fallers, 37 were females and 17 (31.5%) were males. The proportion for frequent fall in the past one year was 72.2% (*n* = 39). Characteristics of fall episode are listed in Table 2. Most of the fall occurred indoors, where only seven (13.0%) occurs outdoors. Time of fall episode usually occurs in the morning (57.4%). Most of the falls episode sustained mild or soft tissue injuries only (79.6%).

### 3.3. Multiple Logistic Regression Analysis of Significant Variables with Their Relation to the Fall among Elderly Diabetes

Table 3 showed the results of multiple logistic regression analysis of potential associated factors for fall among elderly diabetes. The result showed that the predictors for fall among elderly diabetes were age more than 75 years (OR 2.97, 95% CI: 1.12, 7.87), female (OR 2.54, 95% CI: 1.26, 5.11), presence of retinopathy (OR 2.19, 95% CI: 1.02, 4.73), presence of orthostatic hypotension (OR 2.87, 95% CI: 1.22, 6.73) and total balance and gait scoring (OR 0.89, 95% CI: 0.83, 0.95).

### 3.4. Fitness of Multiple Logistic Regression Models

Hosmer-Lemeshow goodness of fit statistic revealed *P* value of 0.692. The sensitivity of the final model was 20%, and the specificity was 90%. The overall percentage was good (81.3%). The area under ROC curve was 0.768.

## 4. Discussion

The prevalence of falls among the elderly with diabetes in this study is lower, compared with previous studies among elderly diabetics [12, 18]. For comparison, a cross-sectional study performed among the elderly attending a diabetes clinic in a university hospital in London found that 39% had suffered at least one fall in the past year [17]. In another community study carried out amongst diabetics from rural areas in North Carolina, the prevalence rate was 43.7% [12].

There was no previous published study on the prevalence of falls among elderly diabetics in Asian countries, thus the comparison of this study with the prevalence of falls among elderly diabetics in Asian countries is not possible. The present study has provided the first set of data on falls among elderly diabetics related to Asians, especially in Malaysia. The prevalence of falls among the general elderly population in Asian countries was noted as almost parallel to this study. It is reported that the prevalence rate in a population-based study in Singapore and Hong Kong was 17.2% [19] and 19.3% [20], respectively. It is noted that the rate of falls is lower in Asian populations compared with US white populations [2]. Thus, given the lower rate of falls for Asians compared with broader populations, we would expect the rate of falls to be lower for Asians in those with diabetes.

The elderly within the age group of more than 75 years old were found to be at a higher risk of falls in this study. Tilling et al. (2006) also found analogous findings in the study of falls among elderly diabetics in the United Kingdom [18]. This correlates many studies that found increasing age is the risk factor for falls among the elderly population [21–23].

Previous studies in western countries have produced mixed findings regarding the issue of gender in the risk of falling among elderly diabetics. Further study is recommended in order to identify the potential causes of the gender disparity. The ELDER study (Evaluating Long-Term Diabetes Self-Management among older women with diabetes) in North Carolina, USA found that men were significantly at greater risk of falls than women [12]. Other studies found no effect on gender [10, 15]. Older women with diabetes have been proven to be at higher risks for falls compared to those without diabetes [6, 8].

Previous researchers found that there is only weak evidence that orthostatic hypotension is a risk factor for falls in the elderly population outside institutions [24, 25]. Schwartz et al. (2008) reported that change in diastolic blood pressure was associated with falls in elderly diabetics but not for systolic blood pressure [11]. From the population-based prospective study by Chu et al. (2005), orthostatic hypotension was not an independent predictor for both a single fall and recurrent falls among elderly people [20].

Almost all studies—with the exception of Miller et al. [3]—have found an association between poor balance and falls in diabetic patients [6, 12]. Poor performances in tests of balance have been related to falls in a previous study of women with diabetes [15].

With respect to eye sight and falls, visual impairment is an important determinant of falling in older persons [26]. In this study, presence of diabetes retinopathy was significantly associated with falls in the elderly diabetes. Schwartz et al. did a prospective study in 446 elderly diabetic in Tennessee and Pennsylvania [11]. In their study, contrast sensitivity, high-contrast distance visual acuity, and depth perception were used as the measurement for visual function. In that study, only contrast sensitivity was associated with increased risks for falls. Not all studies found the association of visual impairment and history of falls in the elderly diabetes. Maurer et al. [15] found that generally diabetes patient has greater visual impairment compared to nondiabetes, but it was not significant. Volpato et al. did a study among diabetic and nondiabetic women and did not find association of visual impairment with increased risk for falls [8].

In univariate analysis (Table 1) fallers had somewhat lower A1C levels than nonfallers. A1C was not retained in the multivariate model, indicating that it was not an important factor associated with falling. Schwartz et al. reported an increased risk of falls among those with low A1C in insulin users but not in those with oral hypoglycemic medications [11]. Tilling et al. reported falls occurred more frequently in those with poor diabetic control [18]. Other studies also did not find any correlation between glucose control and the risk of falls [9]. Thus, although glycemic control is an important goal for other reasons, these results suggest that improved control will not affect fall risks in the short run.

There are few limitations in this study. The retrospective design prone for recall bias and elderly subjects may underestimate the number or seriousness of fall episodes. This is possibly a reason for the lower prevalence rate. Cummings et al. have reported that compared to prospective studies, retrospective studies underestimate the incidence of falls by 13% to 32% depending on the time of period of recall [27]. The cognition of the subjects was not assessed in this study. Hence, older adults with poor memory are more likely to forget about their falls than others.

In conclusion, only diabetic retinopathy, not complications in general, was associated with falls. Other risk factors in addition to retinopathy could identify older diabetic patients at higher risk of falling. These comparisons were made among elderly diabetic patients, not with nondiabetic patients. Elderly diabetics with complications have an augmented risk for falls, and the diabetic clinic review provides the opportunity to screen the elderly diabetes for falls occurrences.

##  Conflict of Interests 

The authors declare that there is no financial and personal relationship with other people or organizations that could inappropriately influence the research.

## Figures and Tables

**Table 1:** Demographic and clinical characteristics of study population.

Variables	Overall (*n* = 288) *n* %	Faller (*n* = 54) *n* %	Nonfaller (*n* = 234) *n* %
Age group (year)			
60–64	118 (41.0)	20 (37.0)	98 (41.9)
65–69	89 (30.9)	9 (16.7)	80 (34.2)
70–74	49 (17.0)	13 (24.1)	36 (15.4)
>75	32 (11.1)	12 (22.2)	20 (8.5)
Sex			
Male	132 (45.8)	17 (31.5)	115 (49.1)
Female	156 (54.2)	37 (68.5)	119 (50.9)
Living arrangement			
Alone	31 (10.8)	7 (13.0)	24 (11.0)
With family	257 (89.2)	47 (87.0)	210 (89.7)
Duration of DM (years)	10.0 ± 11.0	13.3 ± 11.0	9.0 ± 10.3
Barthel's index	98.4 ± 6.7	95.8 ± 11.7	99.0 ± 4.8
Balance and gait scoring	24.1 ± 6.5	20.9 ± 4.5	24.8 ± 6.6
BMI (kg/m^2^)	30.8 ± 4.9	31.2 ± 5.6	31.24 ± 95.4
Hypertension	180 (62.5)	37 (68.5)	144 (61.5)
Nephropathy	44 (15.3)	9 (16.7)	35 (15.0)
Retinopathy	55 (19.1)	17 (31.5)	38 (16.2)
Peripheral neuropathy	139 (48.3)	34 (63.0)	105 (44.9)
Use of insulin	58 (21.0)	15 (27.8)	43 (18.4)
Polypharmacy	232 (80.6)	46 (85.2)	186 (79.5)
Hypoglycemia episode	57 (19.8)	16 (29.6)	41 (17.5)
Orthostatic hypotension	35 (12.2)	13 (24.1)	22 (9.4)
FBS level (mmol/L)	6.9 ± 4.4	6.6 ± 3.9	7.4 ± 4.5
HbA1C level (mmol/L)	8.2 ± 2.4	7.6 ± 2.4	8.3 ± 2.4
LDL level (mmol/L)	3.0 ± 1.2	3.0 ± 1.0	3.1 ± 0.8
Serum creatinine (mmol/L)	115.0 ± 40.7	117.3 ± 34.8	108.0 ± 42.0

**Table 2:** Characteristics of fall episode (*n* = 54).

Characteristics	*n*	Percentage
Location		
Bathroom	21	38.9
Bedroom	12	22.2
Living Room	14	25.9
Others	7	13.0
Timing of fall		
Morning	31	57.4
Afternoon	21	38.9
Evening	2	3.7
Injuries associated with fall		
Mild bruises	43	79.6
Muscle pain	8	14.8
Fracture limb	3	5.5

**Table 3:** Multiple logistic regression analysis of associated factor for fall among elderly diabetes.

Variables	OR^a^	95% CI^b^	Wald^c^	*P* value
Age group >75	2.97	1.12, 7.87	4.82	0.028
Gender Female	2.54	1.26, 5.11	6.83	0.009
Retinopathy	2.19	1.02, 4.73	4.07	0.044
Balance and gait score	0.89	0.83, 0.95	10.17	0.001
Orthostatic hypotension	2.87	1.22, 6.75	5.80	0.015

^a^Adjusted OR.

^b^Confidence interval.

^c^Wald statistic.
